# Physical Activity, Insulin Resistance and Cancer: A Systematic Review

**DOI:** 10.3390/cancers16030656

**Published:** 2024-02-03

**Authors:** Santiago Navarro-Ledesma, Dina Hamed-Hamed, Ana González-Muñoz, Leo Pruimboom

**Affiliations:** 1 Department of Physiotherapy, Faculty of Health Sciences, University of Granada, Campus of Melilla Querol Streel 5, 52004 Melilla, Spain; 2 University Chair in Clinical Psychoneuroimmunology, University of Granada and PNI Europe, 52004 Melilla, Spain; leo@cpnieurope.com; 3 Clinical Medicine and Public Health PhD Program, Faculty of Health Sciences, University of Granada, Av. de la I, 18071 Granada, Spain; dhamed@correo.ugr.es (D.H.-H.); agonzalezm@correo.ugr.es (A.G.-M.); 4 Clinic Ana González, Avenida Hernan Nuñez de Toledo 6, 29018 Malaga, Spain

**Keywords:** insulin resistance, exercise, cancer, metabolic syndrome

## Abstract

**Simple Summary:**

The study aims to assess the impact of physical activity on insulin resistance in cancer patients. It conducted a systematic review of randomized controlled trials published in the last 13 years, using databases like PubMed and Cochrane Library. Among 12 included studies, eight had low bias risk, two had unclear bias risk, and two had high bias risk. Various exercise types were used, making conclusive findings difficult. Seven studies showed improved insulin sensitivity with physical activity, while five showed no significant changes. No adverse effects were reported. Despite evidence of exercise combating insulin resistance in other conditions, the evidence for cancer patients is weak and limited. The review highlights a lack of research on optimal exercise doses, timing, and types for cancer patients. More studies with defined physical activity programs are needed to better understand its role in managing insulin resistance in cancer patients.

**Abstract:**

Introduction: Insulin resistance (IR), a key aspect of type 2 diabetes and a defining characteristic of obesity and its associated conditions, emerges as a mechanistic pathway potentially implicated in cancer pathophysiology. This presents an appealing intervention target for cancer patients. The objective of this study is to conduct a systematic review, examining the scientific evidence regarding the impact of physical activity on modifying insulin resistance in individuals with cancer. Methods: The selection criteria were specific: only randomized controlled clinical trials published in the last 13 years and written in English or Spanish were included. The databases utilized for the search included PubMed, Scopus, Cochrane Library, EBSCO, and WEB OF SCIENCE. The protocol for this review was duly registered in the International Register of Systematic Reviews (CRD42023435002). The final search was conducted on 14 May 2023. Results: The outcomes were assessed using the tool proposed by the Cochrane Handbook to evaluate the risk of bias in the included studies. Among the 12 studies incorporated, 8 demonstrated a low risk of bias, two had an unclear risk of bias, and the remaining two showed a high risk of bias. The variety of exercise types used across all studies was extensive, making definitive conclusions challenging. Physical activity was linked to enhanced insulin sensitivity in seven studies, while five studies showed no significant changes in insulin resistance between the intervention and control groups. Importantly, none of the interventions employed in the included studies exhibited adverse effects on the study participants. Conclusions: The role of exercise as a medicine against insulin resistance has been evidenced in many different studies, mostly related to obesity and cardiovascular diseases. Engaging in physical activity could be a healthy option to combat the effects of insulin resistance in cancer patients, although evidence is weak and limited, according to the results of our systemic review. We further found that literature is lacking at the level of optimal doses, timing, and type of exercise. More studies are needed with more defined PA programs in type and length.

## 1. Introduction

In recent years, various in-depth studies, both in basic and clinical domains, have provided substantial evidence strongly endorsing the idea of a strong connection between obesity, diabetes, and an increased susceptibility to cancer. Insulin resistance (IR), a key aspect of type 2 diabetes and a distinctive characteristic of metabolic syndrome, obesity, and related conditions, emerges as a mechanistic pathway that could potentially be involved in the pathophysiology of cancer. This highlights its potential as an appealing target for intervention in the context of cancer treatment [1]. Several mechanisms have been proposed as contributing to the interplay between IR and cancer at various stages of tumor development. These factors include chronic sustained hyperinsulinemia, insulin receptors (INSRs), insulin-like growth factor 1 receptors (IGF1Rs), and hybrid INSR/IGF1R receptors [1]. Additionally, chronic inflammation, non-coding RNAs (ncRNAs), mitochondrial dysfunction, and the microbiota have also been implicated in this complex relationship [2]. It is crucial to acknowledge that the connection between insulin resistance (IR) and cancer is not solely explained by a single factor at this point.

While there has been extensive research on the role of insulin-like growth factor 1 (IGF1) in various cancers, investigations into the potential impact of insulin itself have been relatively limited [3,4]. Some epidemiological studies have reported an elevated cancer risk in individuals using exogenous long-acting insulin for diabetes therapy [5]. Despite debates surrounding these findings, recent evidence suggests that the use of exogenous insulin is linked to a 20% higher risk of cancer, particularly for liver, pancreatic, bladder, and neurologic tumors [6]. This has sparked increased interest in exploring the role of endogenous insulin in cancer risk.

Although correlational human data have associated elevated insulin levels with various cancer types, establishing a definitive cause-and-effect relationship for any specific cancer type remains elusive [7]. Breaking down cancer types, a notable proportion of liver, pancreatic, breast, endometrial, esophageal, and kidney cancers are associated with obesity and diabetes. Hyperinsulinemia, independent of diabetes, obesity, and metabolic syndrome, has also been correlated with cancer mortality [8].

Insulin is widely recognized as a growth factor for many cell types, including pancreatic cancer cells and their potential precursors. Individuals with hyperinsulinemia exhibit an increased risk of breast, colorectal, prostate, endometrial, liver, and ovarian cancers, regardless of their body mass index (BMI) [9]. Notably, hyperinsulinemia is linked to a two-fold risk of cancer-related death, persisting even in individuals with a normal body weight [10].

However, defining hyperinsulinemia consistently across studies remains challenging due to the absence of a widely accepted insulin concentration threshold. Nevertheless, the robust association between cancer and hyperinsulinemia, supported by strong epidemiological data, underscores the necessity for mechanistic studies to investigate the potential contribution of hyperinsulinemia as a causal factor for cancer [8].

As mentioned earlier, both clinical and epidemiological investigations have indicated a correlation between hyperinsulinemia and an increased risk of cancer morbidity and mortality. Furthermore, animal studies have directly demonstrated that hyperinsulinemia can stimulate tumorigenesis, especially in pancreatic cancer [11]. Utilizing invertebrate models to study hyperinsulinemia-induced cancer offers valuable opportunities for robust screenings, potentially revealing specific molecular mechanisms and paving the way for targeted therapeutics [12].

A study conducted by Sanaki reveals that under normal circumstances (with normal insulin levels), healthy wild-type cells eliminate cancer cells through cell competition. The lower expression of insulin receptors on mutated cancer cells in this model appears to impede cancer progression. Hyperinsulinemia, however, overcomes this restriction, leading to increased survival of cancer cells and the elimination of wild-type cells [12]. Hence, while insulin is essential for maintaining normal life, the adverse effects of hyperinsulinemia emphasize the importance of keeping insulin levels within a healthy range. Exploring effective interventions rooted in lifestyle medicine or therapeutics with mild insulin-suppressing effects offers new possibilities for addressing conditions like obesity, chronic inflammation, and potentially certain cancers. An illustrative example of therapeutic approaches with mild insulin-suppressing effects in cancer involves the use of sodium–glucose cotransporter 2 (SGLT2) inhibitors and/or the induction of a ketogenic state through lifestyle interventions, dietary changes, and the incorporation of medium-chain triglycerides [2].

Simultaneously, it is crucial to identify patients who could derive more substantial benefits from personalized treatments utilizing molecularly targeted drugs. The exploration of such individualized approaches holds the potential to optimize cancer management and enhance patient outcomes [1]. This perspective is supported by an increasing body of evidence, highlighting the advantages of incorporating physical exercise into the management of anticancer treatments and its continuation thereafter, leading to its incorporation into routine practice recommendations.

Physical activity proves effective in addressing issues such as muscle wasting, cardiorespiratory deconditioning (a significant contributor to cancer-related fatigue), and certain symptoms associated with tumors or their treatments, including pain, anxiety, nausea, and sleep disturbances. Robust evidence demonstrates the positive impact of physical exercise on depression, fatigue, anxiety, health-related quality of life (HRQoL), and physical functioning across various cancer types. Moderate evidence also suggests its favorable influence on bone health and sleep quality. Although there is preliminary evidence regarding exercise’s potential benefits for conditions like peripheral polyneuropathy induced by chemotherapy, cognitive function, falls, nausea, pain, sexual function, and treatment tolerance, these areas necessitate further research [13]. These positive effects have been observed in patients with diverse cancer types, both during and after treatment, encompassing breast (predominant), prostate, colorectal, gynecological, head and neck, lung, or hematologic cancers, at early or advanced disease stages [14,15,16,17].

Research supports the idea that exercise interventions in cancer patients, under professional supervision, should be tailored to individual preferences, disease conditions, treatments, and symptoms, ensuring feasibility and safety. This personalized approach is encapsulated by the term “adapted physical activity” (APA). Typically, two types of exercises are combined: endurance training, involving repeated isotonic exercises to enhance aerobic capacity, and resistance training, focusing on muscles against external force. Endurance training improves cardiovascular function, reduces inflammatory responses, and delays heart failure-induced muscle atrophy [18]. It also enhances skeletal muscle mitochondrial activity and promotes mitochondrial biogenesis through increased PGC-1α expression. On the other hand, resistance training targets muscle fiber and its microenvironment, effectively improving muscle mass and function in cancer patients. By normalizing the muscle fiber and its microenvironment through muscle strengthening and aerobic exercises, exercise helps combat conditions like sarcopenia and fatigue, breaking the cycle of deconditioning [18].

Nevertheless, while it is established that exercise is a potent tool in combating insulin resistance and enhancing glucose metabolism, there is a necessity to investigate the variations in insulin resistance among patients with different types of cancer following physical exercise interventions. The objective is to analyze these changes and propose treatment programs tailored to specific biomarkers and their associated physiological responses. Hence, this study aims to conduct a systematic review of scientific evidence regarding the impact of physical activity on modifying insulin resistance in individuals diagnosed with cancer.

## 2. Material and Method

### 2.1. Study Design

We conducted a systematic review adhering to the guidelines outlined by the Preferred Reporting Items for Systematic Review and Meta-Analysis (PRISMA) [19], incorporating randomized controlled trials. The review process followed the PICOS strategy, and the protocol is registered in the International Register of Systematic Reviews under the registration number CRD42023435002. The primary objective of our research was to identify scientific evidence pertaining to the efficacy of physical activity and exercise in altering insulin resistance among individuals diagnosed with cancer. 

### 2.2. Sources Reviewed

The exploration encompassed various databases and computerized platforms, including PubMed, Scopus, Cochrane Library, EBSCO, and WEB OF SCIENCE.

### 2.3. Search Approach

The search strategy employed keywords derived from the Medical Subject Headings (MeSH) thesaurus, such as “exercise,” “insulin resistance,” “metabolic syndrome,” “healthy lifestyle,” and “quality of life/psychology.” Additionally, non-MeSH terms like cancer, nutritional strategies, intermittent fasting, time restriction, insulin resistance index, and homa.ir were incorporated. Boolean operators AND and OR were utilized to combine these terms, with the stipulation that they appear in the title, abstract, or keywords. The final search was conducted on 14 May 2023.

Table 1 outlines the search strategy implemented in PubMed, while Supplementary Table S1 provides a detailed overview of the remaining search strategies.

### 2.4. Inclusion Criteria

The inclusion criteria were defined as follows:

- RCT published within the last 13 years;
- Publications are available in either English or Spanish.

### 2.5. Exclusion Criteria

Exclusion criteria involved studies that did not utilize exercise as an intervention.

### 2.6. Study Selection Process

For storage and removal of duplicates in the identified studies, the Rayyan QCRI program [20] was employed. The selection and identification process involved a meticulous review of titles and abstracts. Subsequently, articles that seemed to meet the inclusion criteria underwent a comprehensive full-text reading.

### 2.7. Data Extraction

Data extraction followed the PICOS strategy, encompassing various characteristics such as author details, publication year, study location, and cancer type. Additionally, information on sample characteristics (size, age, sex), intervention specifics (type, duration, and effects on insulin resistance), and key outcomes (assessment tools, follow-up, and outcomes of the intervention) were extracted.

### 2.8. Risk of Bias Measurement Tool

To evaluate the risk of bias in included studies, the tool recommended by the Cochrane Manual of Systematic Reviews of Interventions was utilized [21]. This tool assesses seven domains, with each domain evaluated as “High risk”(−), “low risk”(+), or “unclear risk”(?). The domains considered for bias assessment include selection bias, performance bias, detection bias, attrition bias, reporting biases, and other sources of bias where unaddressed biases of significance may be noted.

### 2.9. Quality of the Evidence

The Grading of Recommendations, Assessments, Development, and Evaluation (GRADE) [22] tool was employed to gauge the quality of evidence for the outcomes reported in the included studies. This system characterizes evidence quality by the level of confidence in the adequacy of effect estimates to formulate recommendations. The assessment takes into account the study risk of bias, inconsistency, imprecision, publication bias, indirect results, and other factors influencing evidence quality.

## 3. Results

### 3.1. Study Identification and Selection Process

**Figure 1:**
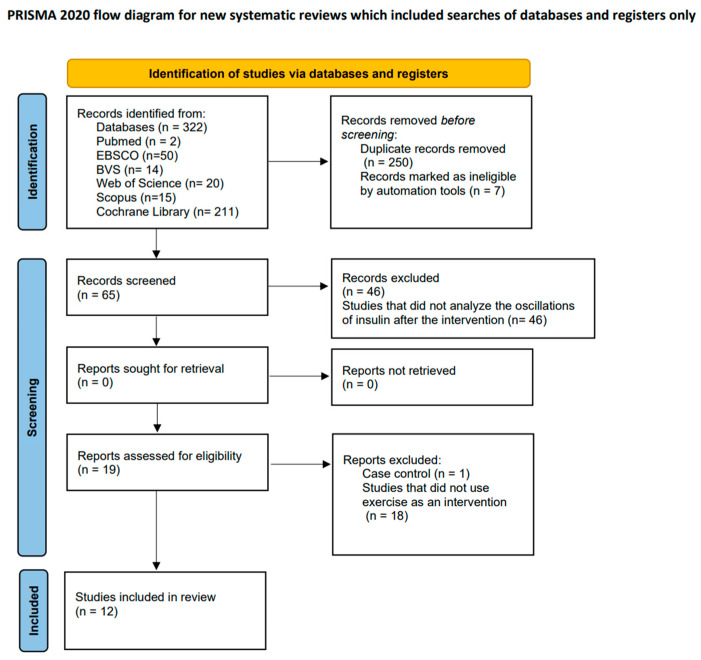
Flow diagram illustrating the study selection process.

During the study identification and selection phase, a total of 322 articles were initially located across various computerized databases. Following the elimination of duplicates, 257 studies underwent a review of their titles and abstracts to determine compliance with the inclusion criteria. Subsequently, a total of 19 studies met these criteria, leading to a comprehensive full-text evaluation. Ultimately, after the full-text assessment, 12 studies [23,24,25,26,27,28,29,30,31,32,33,34], aligning with the inclusion criteria, were incorporated into this systematic review. The study selection process is visually represented in the flow diagram provided (Figure 1).

### 3.2. General Characteristics of the Selected Studies

The studies included in this systematic review consisted of eight randomized controlled articles [23,25,26,27,29,30,31,34], two pilot trials [24,28], and two single-arm studies [32,33]. The publication period ranged from 2010 to 2023, with 2013 having the highest number of published articles [25,26,27].

Of these 12 studies, one was conducted in the United Kingdom [24], another in Finlandia [25], one in Osaka [26], two in Denmark [27,28], two in Italy [30,34], one in Australia [23] and the remaining four studies in the United States [30,31,32,34]. 

The sum of the sample sizes from the nine included studies comprises a total of 1389 individuals. It is worth noting that none of the interventions used in all the included studies showed any adverse effects in the study participants.

Regarding gender, the study population consists of both men and women. Age varied with a range of 18 years of age, and patients suffered from breast cancer, prostate cancer, and liver cancer (Table 2).

### 3.3. Risk of Bias

Most studies exhibited a high risk of bias across various domains [23,24,25,26,27,28,29,30,31,32,33,34]. Specifically, the blinding of participants and personnel, along with the blinding of assessors, indicated a high risk of bias in the articles by Nobes et al. and Hvid et al. [24,25,26,27,28].

Table 3 provides an overview of the risk of bias in all included articles. Different colors in the table signify the methodological quality of the studies: High risk (red), unclear risk (yellow), and low risk of bias (green). 

### 3.4. Intervention Characteristics

The included studies investigated the impact of physical activity on the metabolic profiles of cancer patients through plasma tests conducted before and after the intervention.

#### 3.4.1. Breast Cancer

Etme et al. [25]: This study examined the impact of obesity and physical activity on the health and well-being of breast cancer patients. Three groups were established based on participants’ body mass index (G1: BMI < 25, G2: BMI 25–30, G3: BMI ≥ 30). The study lasted for one year, and physical activity was measured using a two-week prospective diary. Cardiovascular performance was assessed through a 2 km walking test.

Bruno E et al. [29]: This study evaluated the effect of aerobic exercise intervention on insulin parameters and body composition in non-obese women without insulin resistance. Two groups were established in this trial: a control group where women received recommendations to engage in physical activity for at least 30 min daily and an intervention group where, in addition to these recommendations, women participated in a supervised exercise program twice a week for 3 months.

Dieli-Conwright C et al. [30]: This study consists of two groups. An intervention group where aerobic and resistance exercises were performed three times a week for a treatment duration of 16 weeks. In this group, serum biomarkers were assessed at the beginning, after the intervention (16 weeks), and at 3 months of follow-up. The other group is a control group composed of patients receiving standard care. Physical activity/dietary assessments were evaluated using a validated questionnaire administered by an interviewer. Participants wore a heart rate monitor, and the sessions were supervised by a trainer.

Viskochi et al. [32]: The objective of this study was to assess exercise-induced changes in insulin concentrations and their associations with biomarkers relevant to breast cancer. Fifteen postmenopausal breast cancer survivors underwent a twelve-week aerobic exercise program, with sessions scheduled three or four days per week, lasting one hour each. Insulin levels were determined using peak glucose and insulin values and the area under the insulin curve during a five-minute oral glucose tolerance test. 

Pistelli et al. [33]: The aim of this study was to assess the impact of a twelve-month supervised intervention on body mass change in breast cancer survivors at high risk of being overweight. It is a single-arm study where the intervention focused on an individualized Mediterranean diet with a low glycemic index and physical activity involving both moderate-intensity aerobic and resistance exercises, conducted 3–5 times per week.

Glucose and insulin levels were assessed using fasting blood samples.

In the study conducted by Dieli-Conwright et al. [34], the aim was to investigate the impact of a 16-week exercise intervention on the self-reported sleep quality of breast cancer survivors. Additionally, the study sought to assess whether alterations in sleep patterns were correlated with cardiometabolic biomarkers. Two groups were established, consisting of overweight breast cancer survivors. There was a control group where patients received standard care and an intervention group where patients received exercise. The 16-week exercise intervention group included both aerobic and resistance exercises. 

#### 3.4.2. Prostate Cancer

Galvao et al. [23] conducted a trial with two arms—an experimental arm and a control arm. Participants in the experimental arm underwent combined progressive resistance and aerobic exercises, engaging in training sessions twice a week over a span of 12 weeks. The aerobic component of the program included 15–20 min of cardiovascular exercises such as cycling, walking, and/or jogging. Fasting blood samples were collected to measure insulin and glucose levels.

In the study by Nobes et al. [24], two groups were established: a control group receiving androgen deprivation therapy and an intervention group receiving androgen deprivation therapy along with metformin, a low glycemic index diet, and exercise for a duration of 6 months. All patients underwent assessments before the treatment and at the 6-month mark. Blood analyses were conducted at 3 months.

Hvid et al. [27] explored whether resistance training improves insulin sensitivity and body composition in prostate cancer patients undergoing androgen deprivation therapy. The study involved two groups—an experimental group of prostate cancer patients receiving androgen deprivation therapy and engaging in a resistance training program and a control group of healthy individuals solely undergoing the resistance program. The resistance program for both groups lasted 12 weeks, was organized three times a week, and included a 5 min warm-up, 35 min of interval training, and a 5 min cool-down. Various training programs were implemented to ensure motivation and adherence.

In the study by Hvid et al. [28], the effect of a 2-year home-based resistance training intervention on body composition, biomarkers, and prostate-specific antigen doubling time was assessed. Two groups were formed: a control group encouraged to maintain their daily routines and exercise patterns and an experimental group where a supervised training program was implemented. Subjects in the training group performed unsupervised resistance training three times a week for 45 min over 24 months, targeting a heart rate corresponding to the desired percentage of maximal oxygen consumption (VO2max). A 2-h 75 g oral glucose tolerance test (OGTT) was conducted after an overnight fast, with blood samples collected at 0, 30, 60, 90, and 120 min for plasma glucose and plasma insulin measurements.

Freedland et al. [31] designed a study to compare a low-carbohydrate diet intervention combined with 30 min of daily walking, 5 days a week, to a control group advised to maintain their regular diet and exercise patterns. 

#### 3.4.3. Liver Cancer

Kaibori et al. [26] aimed to assess the effects of exercise therapy on patients with hepatocellular carcinoma undergoing hepatectomy. The study involved two groups: a control group following a specific diet and an experimental group participating in an exercise program in addition to the prescribed diet. Before commencing the exercise program, patients in the experimental group underwent a cardiopulmonary exercise test on an ergometric bicycle, utilizing an incremental protocol. The exercise program consisted of three 60 min sessions per week. Each session comprised 5 min of stretching exercises, 30 min of walking at an intensity based on each patient’s anaerobic threshold, 20 min of targeted stretching exercises, and a 5 min cooldown with stretching. 

Table 4 shows the intervention characteristics in detail.

### 3.5. Results from each study

#### 3.5.1. Breast Cancer

Elme et al. [25]: In the experimental group, a higher fasting insulin level was observed (*p* = 0.0098). The prevalence of metabolic abnormalities was higher in obese individuals (68%), overweight patients (39%), and patients with normal weight (11%), with physical activity and the risk of breast cancer being associated with insulin resistance. In this study, obesity and physical inactivity were linked to hyperinsulinemia.

Bruno E et al. [29]: In terms of insulin resistance parameters, the experimental group showed a non-significant decrease in insulin levels and the HOMA index, whereas the control group exhibited a non-significant increase in these parameters. These results suggest that a structured 3-month exercise program improves insulin levels, HOMA index, and body composition parameters in breast cancer survivors without insulin resistance.

Dieli-Conwright C et al. [30]: Insulin improved in the exercise group compared to the control group (*p* = 0.002), and at the 3-month follow-up, the improvement continued (*p* < 0.01). The combination of aerobic and resistance exercises proved to be effective in mitigating metabolic syndrome in sedentary, overweight breast cancer survivors.

Viskochil et al. [32]: In this study, no significant pre- and post-exercise changes in glucose and insulin levels were observed. Physical training had limited overall benefits on insulin concentrations after glucose administration in breast cancer survivors.

Pistelli et al. [33]: In this study, significant differences were found in blood glucose, insulin, and testosterone levels. Therefore, the integration of exercise programs along with dietary interventions is suggested for breast cancer patients.

Dieli-Conwright et al. [34] investigated the correlation between changes in patient-reported sleep quality and alterations in body composition, cardiometabolic biomarkers, or systemic inflammation. The study found a positive correlation between changes in sleep quality and total cholesterol as well as adiponectin (with correlation coefficients of 0.74 and 0.75, respectively; *p* < 0.01). Moreover, the change in patient-reported sleep quality exhibited a negative correlation with HDL (correlation coefficient: −0.83; *p* < 0.01). Positive correlations were observed with all seven sleep components except for sleep medication (*p* > 0.05; correlation coefficient >0.50). Notably, leptin did not show significant associations with any sleep component (correlation coefficient ≤ 0.40; *p* > 0.05).

#### 3.5.2. Prostate Cancer

Galvao et al. [23]: No significant differences were observed between groups for glucose and insulin biomarkers; however, the quality of life increased in the experimental group. There were no adverse effects during the exercise sessions.

Nobes J et al. [24]: The changes in biochemical markers of insulin resistance over time did not differ significantly between the groups.

Hvid et al. [27]: A significant effect was achieved in reducing fasting glucose concentrations (*p* < 0.05).

Hvid et al. [28] reported a decrease in fasting plasma glucose levels after 24 months of training (*p* = 0.05), while no significant changes were observed in the control group. The alteration in the area under the insulin curve after 24 months showed a tendency to be different between the two groups (*p* = 0.06); however, no notable changes were observed in fasting plasma insulin levels, the area under the plasma glucose curve, or insulin sensitivity indices in either of the groups. Freedland et al. [32]: In this study, walking did not improve insulin sensitivity at the 6-month mark of the trial, although a secondary analysis indicated that the combination of a low-carbohydrate diet and walking reduced insulin resistance during the study. This supports the need for broader intervention studies to mitigate the alterations caused by androgen deprivation therapy.

#### 3.5.3. Liver Cancer

Kaibori et al. [26]: In the exercise group, fasting serum insulin and HOMA-IR showed significant decreases at 3 and 6 months after liver cancer surgery compared to the diet group.

Table 5 and Table 6 show the results in detail.

### 3.6. Grade System

The quality of evidence in this systematic review is low. Assessments have heavily relied on the risk of bias in the trials and the imprecision of their results.

The quality of the evidence is low in studies with only one arm.

For more detailed information, see Supplementary Table S2.

## 4. Discussion

The purpose of our study was to find evidence for the use of PA as a remedy against insulin resistance in patients suffering from different types of cancer. Only 12 studies complied with our inclusion criteria, which itself was a surprisingly low number in view of the effects of PA in patients suffering from metabolic syndrome, diabetes type 2, and many other maladies [23,24,25,26,27,28,29,30,31,32,33,34]. As patients suffering from metabolic syndrome and/or diabetes type 2 patients have an increased risk for global cancer development, we expected to find more studies. Next to the fact that only 12 studies were found, the overall quality of the 12 studies was low and followed by a high disparity in results. [23,24,25,26,27,28,29,30,31,32,33,34]. When comparing the outcome of PA on insulin resistance in different kinds of cancer patients, women with breast cancer seem to benefit the most, whereas patients with pancreatic cancer hardly benefit from PA when searching for improvement of IR in those patients. In patients with liver cancer, insulin resistance improves with PA, although only one study has been included in our systematic review. The overall disparity in PA on IR results between the different cancer types and even between studies of the same cancer type can partly be explained by the low-quality evidence of the included studies but also because of different types of intervention and exercise protocol. Moderate and intensive exercise both improve insulin sensitivity in obese sedentary people after a 12-week program, four sessions per week independent of weight loss [35]. In this study, it was clear that the direct effects of exercise itself were responsible for the increase in insulin sensitivity and not based on earlier training effects [35]. A frequently cited paper by Bird [36] describes the different pathways with which PA increases insulin sensitivity. Exercise increases arterial blood circulation in active muscles, which increases insulin sensitivity, and the same holds for the GLUT4 increase after muscle activity [36]. Their review ends with the notion that the best way to improve insulin sensitivity is to combine PA with dietary interventions [36]. 

D’Alonzo et al. [37] investigated the combined effects of PA and weight loss measurements on insulin and insulin resistance in breast cancer survivors compared with exercise alone and a control group [37]. The participants treated with weight loss and combined measurements exhibited a significant decrease in HOMA-2, IR, beta-cell function, and c-peptide levels compared with the control group. Weight loss alone was already responsible for >10% improvement in insulin sensitivity [37]. Our results related to the impact of PA on IR in cancer patients are much more ambiguous. Although combined interventions to reduce insulin resistance seem more effective in patients suffering from diseases other than cancer, our results do not support these findings. In the study of Nobes [24], the intervention group was treated with androgen deprivation therapy along with 6 months of metformin, a low glycemic index diet, and exercise; no significant changes in insulin resistance of these interventions were achieved. All studies included in our systematic review use aerobic exercise as a basic intervention. Low-intensity PA is less effective than moderate and high-intensity PA in sedentary people, although every form of exercise is beneficial for the improvement of insulin sensitivity in sedentary people [38,39]. Hyperinsulinemia is a hallmark of insulin resistance [40], and hyperinsulinemia increases the production of IGF-1 significantly, which is related to the initiation and progression of multiple cancer types. In a study by Murphy et al. [41], it was shown in a group of 430,000 patients that IGF-1 is associated with multiple cancer types, including breast cancer and thyroid cancer, and that IGF-1 can even be considered causal to different cancer types [41]. The probability that insulin resistance in people with cancer can be resolved solely with exercise and in the light of all ‘strategies’ used by tumor cells to prevail and progress is at least doubtful. Using exercise as complementary is definitely interesting in treating people with several types of cancer. Nevertheless, exercise alone, as evidenced in this review, is not effective enough and is a mono-intervention. Other interventions that influence inflammation, immune suppression, and the tumor micro-environment are probably needed to make exercise more effective, not only related to insulin resistance but also for the overall outcome in patients suffering from cancer.

### 4.1. Practical Application

Exercise has been shown to be one of the lifestyle factors that reduce the risk of developing cancer, leading to a biochemical response that subsequently lowers plasma insulin levels [42]. Exercise as medicine should undoubtedly be part of an integrative treatment protocol for people suffering from cancer. Studies should focus on the intensity, timing, dosage, and type of exercise in different types of cancer and types of patients. Exercise programs should be personalized and adapted to the patient, the type of cancer, and the context of the patient’s circumstances and not vice versa. 

**Figure 2:**
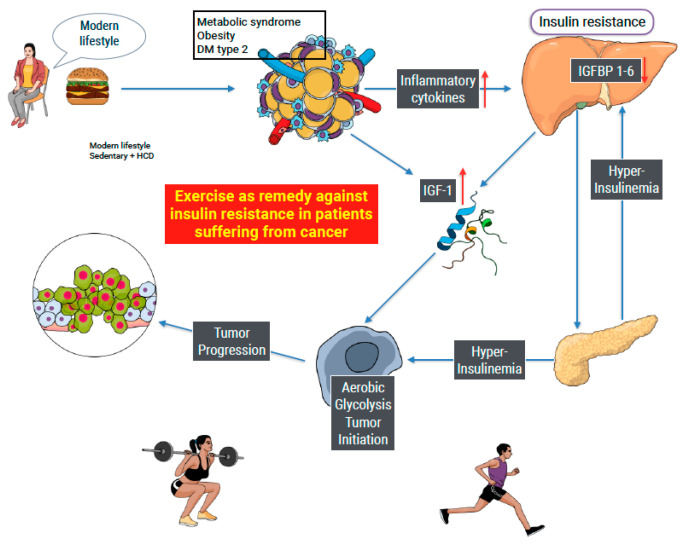
Modern lifestyle as an example of the initiation of cancer. A high-calorie diet and sedentary behavior lead to hypertrophy of white adipose tissue, inducing both inflammation and the production of IGF-1. Inflammatory cytokines produce insulin resistance and a compensatory increase in insulin production (hyperinsulinemia). Insulin augments the production of IGF-1, limiting the production of IGF 1–6, leaving IGF-1 free. Both insulin and IGF-1 are able to cause a metabolic shift of cell metabolism from OxPhos to aerobic glycolysis, known as the Warburg effect. The metabolic shift, when maintained in time, can cause tumor initiation and progression. Exercise is known to improve insulin sensitivity in sedentary people. Our study searched for similar effects in people suffering from cancer with ambiguous results.

Figure 2 shows how modern life causes chronic inflammation systemic insulin resistance (visualized in the liver) and subsequence leads to hyperinsulinemia, an increase in free IGF-1, and possible induction of aerobic glycolysis (Warburg effect) in susceptible organs as an initiator of tumor development. Long-term chronic inflammation and increased IGF-1/Insulin production can lead to cancer progression. Our study searched for evidence of the influence of PA on insulin resistance in people suffering from cancer. The Figure summarizes the way insulin and IGF-1 affect cell metabolism and possible cancer development. 

### 4.2. Study Limitation

Several limitations should be emphasized. First, a surprisingly small number of studies found that analyzed insulin sensitivity in cancer patients who engaged in physical activity as an intervention to improve metabolic pathways. Secondly, the low quality of evidence of the studies that were included in our systematic review. Third, the interventions showed low coherence and poor description. Therefore, hard-end conclusions are impossible to give. Nevertheless, our results show that exercise influences insulin resistance in some cancer patients and with some protocols, but generalizing is not possible.

### 4.3. Prospective

After reviewing and analyzing the results, it can be concluded that there is a lack of scientific evidence regarding which type of exercise is most optimal for improving insulin resistance in cancer patients. It is also important to implement a comprehensive treatment approach alongside nutritional strategies since, according to the literature, the combination of diet and natural processes plays a significant role in the metabolic pathways affecting cancer [43]. In a systematic review by Navarro-Ledesma S et al. [44], it is suggested that cancer should be considered systemic and multifactorial, meaning that the success of treatment should be multifactorial and systemic [44].

Therefore, the following lines of research are proposed:

Conducting randomized controlled trials (RCTs) that combine physical activity and nutritional strategies to improve insulin sensitivity in individuals with cancer. Additionally, the impact of lifestyle interventions, exercise dosage, and multimodal approaches on cancer location, considering toxicities, is crucial for comprehensive understanding.

## 5. Conclusions

This systematic review presented mixed evidence on the impact of exercise interventions on insulin sensitivity in cancer patients. While some studies demonstrated positive effects, others did not show significant improvements, emphasizing the need for standardized protocols in future research. This systematic review shows that engaging in physical activity is a healthy option to combat the effects of insulin in cancer patients. Combining aerobic and resistance exercises seems to improve insulin sensitivity, body composition, and overall quality of life in cancer survivors. The heterogeneity in exercise interventions and cancer types highlights the importance of tailoring exercise programs to specific cancer conditions for optimal outcomes.

## Figures and Tables

**Table 1:** Search Strategy.

Platform	Search Strategies
PubMed	((“Neoplasms”[Mesh]) AND (“Diet”[Mesh] OR “Fasting”[Mesh] OR “Caloric Restriction”[Mesh] OR “Diet, Ketogenic”[Mesh] OR “exercise”[Mesh]) AND (“insulin resistance”[Mesh] OR “metabolic syndrome”[Mesh]) AND (“Healthy Lifestyle”[Mesh] OR “Quality of Life/psychology”[Mesh])) OR ((“Cancer”[tw] OR “neoplasms”[tw]) AND (“Fasting”[tw] OR “Caloric Restriction”[tw] OR “Diet, Ketogenic”[tw] OR “Nutritional strategies” [tw] OR “intermittent fasting”[tw] OR “time restriction” [tw] OR “exercise” [tw]) AND (“insulin resistance”[tw] OR “insulin resistance index”[tw] OR “metabolic syndrome”[tw] OR “homa-ir”[tw]) AND (“Healthy Lifestyle”[tw] OR “Quality of Life/psychology”[tw]))

**Table 2:** Characteristics of the included studies.

Author	Year	Study Type	Country	Cancer	Sample	Gender	Age (Years)
Galvao et al. [23]	2010	RCC	Australia	Prostate Cancer	*n* = 57	Male	69.5 ± 7.3
Nobes J et al. [24]	2011	PE	UK	Prostate Cancer	*n* = 40	Male	69.5
Elme et al. [25]	2013	RCC	Finlandia	Breast Cancer	*n* = 537	Female	52
Kaibori et al. [26]	2013	RCC	Osaka	Liver Cancer	*n* = 51	36 male	69.7
						15 female	
Hvid et al. [27]	2013	RCC	Denmark	Prostate Cancer	*n* = 21	Male	67.8
Hvid 2 et al. [28]	2015	PE	Denmark	Prostate Cancer	*n* = 17	Male	69.8
Bruno E et al. [29]	2016	RCC	Italy	Breast Cancer	*n* = 38	Female	56.5 ± 6.8
Dieli-Conwright C et al. [30]	2018	RCC	EEUU	Breast Cancer	*n* = 100	Female	53 ± 10.4
Freedland et al. [31]	2019	RCC	EEUU	Prostate Cancer	*n* = 42	Male	66
Viskochil et al. [32]	2020	E	EEUU	Breast Cancer	*n* = 15	Female	59.9 ± 9.2
Pistelli et al. [33]	2021	E	Italy	Breast Cancer	*n* = 98	Female	>18
Dieli-Conwright C 2 et al. [34]	2022	ECC	EEUU	Breast Cancer	*n* = 418	Female	52 ± 10.4

Abbreviations: ECC: Controlled clinical trial, PE: Pilot Study, E: One ArmTrial, UK: United Kingdom, EEUU: United States.

**Table 3:** Risk of bias.

	Galvao et al. [23]	.Nobes J et al. [24]	Elme et al. [25]	Kaibori et al. [26]	Hvid et al. [27]	Hvid 2 et al. [28]	Bruno E et al. [29]	Dieli-Conwright C et al. [30]	Freedland et al. [31]	Viskochil et al. [32]	Pistelli et al. [33]	Dieli-Conwright C 2 et al. [34]
Proper sequence generation (selection risk)	+	+	+	+	+	+	+	+	+	-	-	+
Selection hiding (selection bias)	+	+	+	+	+	+	+	+	+	-	-	+
Blinding of participants and staff (implementation bias)	+	+	+	+	+	+	+	+	+	-	-	+
Blinding of outcome evaluators (detection bias)	+	-	+	+	+	-	+	+	+	-	-	+
Incomplete results data (wear bias)	+	-	+	+	+	-	+	+	+	-	-	?
Selective reporting of results (notification bias)	+	+	+	+	+	+	+	+	+	-	-	+
Other sources of bias	?	?	?	?	?	?	?	?	+		?	?

Abbreviations: (+): Low risk of bias (-): High risk of bias (?): Unclear risk of bias. High risk (red), unclear risk (yellow), and low risk of bias (green).

**Table 4:** Intervention Characteristics.

Author	Year	Type of Intervention	Groups			Duratión Intervention	Frequency Intervention	Variable	Measuring Instrument
Galvao et al. [23]	2010	Aerobic and resistance exercises	GE = 29 GC = 28			12 weeks	2 times/week	Insulin	
Nobes J et al. [24]	2011	Exercise program	GE = 20	GC = 20		24 weeks	Not shown	Insulin	HOMA-IR
Elme et al. [25]	2013	Exercise program	G1:IMC < 25	G2:IMC 25–30	G3:IMCC ≥ 30	48 weeks	Not shown	Insulin	HOMA-IR
Kaibori et al. [26]	2013	Aerobic and resistance exercises	GE = 26	GC = 25		24 weeks	3 times/week	Insulin	HOMA-IR
Hvid et al. [27]	2013	Resistance exercises	GE = 9	GC = 10		12 weeks	3 times/week	Insulin	HOMA-IR
Hvid2 et al. [28]	2015	Resistance Training at home	GE = 12	GC = 7		96 weeks	3 times/week	Insulin	HOMA-IR
Bruno E et al. [29]	2016	Aerobic exercises	GE = 28	GC = 20		12 weeks	2 times/week	Insulin	HOMA-IR
Dieli-Conwright C et al. [30]	2018	Aerobic and resistance exercises	GE = 50	GC = 50		16 weeks	3 times/week	Insulin	HOMA-IR
Freendlan et al. [31]	2019	Walk	GE = 20	GC = 22		24 weeks	5 times/week	Insulin	HOMA-IR
Viskochil et al. [32]	2020	Supervised aerobic exercise program	G1 = 15			12 weeks	3–4 times/week	Insulin	Oral glucose tolerance test.
Pistelli et al. [33]	2021	Aerobic and resistance exercises	G1 = 98			48 weeks	3–5 times/week	Insulin	HOMA IR
Dieli-Conwright C 2 et al. [34]	2022	Aerobic and resistance exercises	GE = 50	GC = 50		16 weeks	3 times/week	Insulin	HOMA-IR

Abbreviations: GE: experimental group, GC: Control group.

**Table 5:** Initial Insulin Levels.

Author	Variable	Before Treatment	
Galvao et al. [23]	Insulin	GE:9.4 (7.2) GC:10.0 (5.7)	
Nobes J et al. [24]	Insulin	GE:107.0 (160.0)	GC = 61.69 (40.0)
Etme et al. [25]			
Kaibori et al. [26]	Insulin	GE = 8.56, 5.8	GC = 9.16, 4.5
Hvid et al. [27]	Insulin	GE93.4	GC = 58.6
Hvid2 et al. [28]	Insulin	GE = 48.0 ± 28.5	GC = 42.0 ± 23.0
Bruno E et al. [29]	Insulin	GE = 7.9 ± 4.2	GC = 6.60 ± 3.1
Dieli-Conwright C et al. [30]	Insulin	GE = 11.1 (8.9)	GC = 12.3 (7.1)
Freedland et al. [31]	Insulin	GE = −16.9 (−42.6, −3.2)	GC = 7.0 (−9.6, 49.7)
Viskochil et al. [32]	Insulin	G1 = 11.4 ± 5.4	
Pistelli et al. [33]	Insulin	G1 = 3.78 (0.06)	
Dieli-Conwright C 2 et al. [34]	Insulin	−	−

Mean (SD).

**Table 6:** Insulin levels after treatment.

Author	Variable	After Treatment		*p*
Galvao et al. [23]	Insulin	GE: 10.2 (7.4) GC: 10.9 (7.1)		*p* = 0.435
Nobes J et al. [24]	Insulin	GE = 92.4 (153.3)	GC = 66.6 (53.7)	*p* > 0.5
Etme et al. [25]				*p* = 0.0098
Kaibori et al. [26]	Insulin	GE = 5.86, 2.8	GC = 10.76, 4.6	*p* = 0.193
Hvid et al. [27]	Insulin	GE = 78.3	GC = 56.0	*p* = 0.030
Hvid2 et al. [28]	Insulin	GE = 44.5 ± 23.5	GC = 42.3 ± 21.3	*p* = 0.05
Bruno E et al. [29]	Insulin	GE = 6.7 ± 3.1 (*p* = 0.8)	GC = 7.43 ± (4.5) (*p* = 0.17)	*p* = 0.4
Dieli-Conwright C et al. [30]	Insulin	GE = 6.8 (1.3)	GC = 15.1 (6.4)	*p* = 0.002
Freedland et al. [31]	Insulin	GE = 7.8 (−35.2, 29.1)	GC = 26.2 (−1.2, 80.7)	*p* = 0.11
Viskochil et al. [32]	Insulin	G1 = 11.6 ± 5.1		*p* = 0.79
Pistelli et al. [33]	Insulin	G1 = 10.51 (0.17)		*p* = 0.923
Dieli-Conwright C 2 et al. [34]	Insulin	-	-	*p* < 0.05

Mean (SD).

## Supplementary Materials

The following supporting information can be downloaded at https://www.mdpi.com/article/10.3390/cancers16030656/s1, Table S1. Search strategies; Table S2. GRADE System.
