# Supplementary material for: Physical Activity, Insulin Resistance and Cancer: A Systematic Review
Source: Cancers (Basel). 2024 Feb 3;16(3):656. doi: 10.3390/cancers16030656 (PMC10854631; doi:10.3390/cancers16030656)
Supplement: Supplementary file 1 [file cancers-16-00656-s001.zip › cancers-2694828-supplementary.pdf]

Physical Activity, Insulin Resistance and Cancer: A Systematic Review

Table S1. Search strategies.

Platform	Search Strategies
PubMed	((“Neoplasms”[Mesh]) AND (“Diet”[Mesh] OR "Fasting"[Mesh] OR "Caloric Restriction"[Mesh] OR "Diet, Ketogenic"[Mesh] OR “exercise”[Mesh]) AND ("insulin resistance"[Mesh] OR “metabolic syndrome”[Mesh]) AND ("Healthy Lifestyle"[Mesh] OR "Quality of Life/psychology"[Mesh])) OR ((“Cancer”[tw] OR “neoplasms”[tw]) AND ("Fasting"[tw] OR "Caloric Restriction"[tw] OR "Diet, Ketogenic"[tw] OR “Nutritional strategies” [tw] OR "intermittent fasting"[tw] OR “time restriction” [tw] OR “exercise” [tw]) AND ("insulin resistance"[tw] OR “insulin resistance index”[tw] OR “metabolic syndrome”[tw] OR “homa-ir”[tw]) AND ("Healthy Lifestyle"[tw] OR "Quality of Life/psychology"[tw]))
SCOPUSS	((“Cancer” OR “neoplasms”) AND ("Fasting" OR "Caloric Restriction" OR "Diet, Ketogenic" OR “Nutritional strategies” OR "intermittent fasting" OR “time restriction” OR “exercise”) AND ("insulin resistance" OR “insulin resistance index” OR “metabolic syndrome” OR “homa-ir”) AND ("Healthy Lifestyle" OR "Quality of Life/psychology"))
LILACS IBECS (BVS)	Tw ((“Cancer” OR “neoplasms”) AND ("Fasting" OR "Caloric Restriction" OR "Diet, Ketogenic" OR “Nutritional strategies” OR "intermittent fasting" OR “time restriction” OR “exercise”) AND ("insulin resistance" OR “insulin resistance index” OR “metabolic syndrome” OR “homa-ir”) AND ("Healthy Lifestyle" OR "Quality of Life/psychology"))
Cochrane Library	#1 cancer 218648<br>#2 neoplasms 91859<br>#3 fasting 41077<br>#4 caloric restriction 2721<br>#5 diet, ketogenic 710<br>#6 nutritional strategies 2303

Table S1. Search strategies.

Platform	Search Strategies
	#7 intermittente fasting 53<br>#8 time restriction 7939<br>#9 exercise 126680<br>#10 insulin resistance 17568<br>#11 insulin resistance index 7331<br>#12 metabolic syndrome 12797<br>#13 homa.ir 4319<br>#14 healthy lifestyle 7051<br>#15 quality of life 160056<br>#16 MeSH descriptor: [Neoplasms] explode all trees 110900<br>#17 MeSH descriptor: [Neoplasms] explode all trees 110900<br>#18 MeSH descriptor: [Diet] explode all trees 25582<br>#19 MeSH descriptor: [Fasting] explode all trees 4019<br>#20 MeSH descriptor: [Caloric Restriction] explode all trees 1137<br>#21 MeSH descriptor: [Exercise] explode all trees 38046<br>#22 MeSH descriptor: [Insulin Resistance] explode all trees 8744<br>#23 MeSH descriptor: [Metabolic Syndrome] explode all trees 2418<br>#24 MeSH descriptor: [Healthy Lifestyle] explode all trees 1358<br>#25 MeSH descriptor: [Quality of Life] explode all trees 43301<br>#26 ((#17 OR #18) AND (#19 OR #20 OR #21) AND (#22 OR #23) AND (#24 OR #25)) OR ((#1 OR #2) AND (#3 OR #4 OR #5 OR #6 OR #7 OR #8 OR #9) AND (#10 OR #11 OR #12 OR #13) AND (#14 OR #15)) 456
EBSCO	MH ((“Neoplasms”) AND (“Diet” OR "Fasting" OR "Caloric Restriction" OR "Diet, Ketogenic" OR “exercise”) AND ("insulin resistance" OR “metabolic syndrome”) AND ("Healthy Lifestyle" OR "Quality of Life/psychology")) OR ((“Cancer” OR “neoplasms”) AND ("Fasting" OR "Caloric Restriction" OR "Diet, Ketogenic" OR “Nutritional strategies” OR "intermittent fasting" OR “time restriction” OR “exercise”) AND ("insulin resistance" OR “insulin resistance index” OR “metabolic syndrome” OR “homa-ir”) AND ("Healthy Lifestyle" OR "Quality of Life/psychology"))
Web of science	TS=((“Cancer” OR “neoplasms”) AND ("Fasting" OR "Caloric Restriction" OR "Diet, Ketogenic" OR “Nutritional strategies” OR "intermittent fasting" OR “time restriction” OR “exercise”) AND ("insulin resistance" OR “insulin resistance index” OR “metabolic syndrome” OR “homa-ir”) AND ("Healthy Lifestyle" OR "Quality of Life/psychology"))

Table S2. GRADE System.

Author(s):

Question: [intervention] compared to [comparison] for [health problem and/or population]

Setting:

Bibliography:

Certainty assessment							Impact	Certainty	Importance
№ of studies	Study design	Risk of bias	Inconsistency	Indirectness	Imprecision	Other considerations			
**Insulin and Cancer (follow-up: range 12 weeks to 48 weeks; assessed with: Homa IR)**									
12	randomised trials	serious[a]	serious[b]	serious	not serious	dose response gradient	7 studies showed significant improvements in the experimentsl group compared to the control group, while the remaining 5 studies found no between group differences. [c]	⊕⊕◯◯ Low	IMPORTANT

CI: confidence interval0

Explanations

a. Due to lack of blinding of assessors in two studies and lack of blinding and random sequencing in single arm studies

b. 7 studies found significant improvements in metabolic biomarkers, while 5 studies found no significant between-group improvements.

c. 7 studies showed significant improvements in the experimental group compared to the control, while the remaining 5 studies found no between-group differences.
